# Low-intensity pulsed ultrasound rescues insufficient salivary secretion in autoimmune sialadenitis

**DOI:** 10.1186/s13075-015-0798-8

**Published:** 2015-10-07

**Authors:** Minami Sato, Shingo Kuroda, Karima Qurnia Mansjur, Ganzorig Khaliunaa, Kumiko Nagata, Shinya Horiuchi, Toshihiro Inubushi, Yoshiko Yamamura, Masayuki Azuma, Eiji Tanaka

**Affiliations:** Department of Orthodontics and Dentofacial Orthopedics, Institute of Biomedical Sciences, Tokushima University Graduate School, Tokushima, Japan; Genetic Disease Program, Sanford Children’s Health Research Center, Sanford-Burnham Medical Research Institute, La Jolla, CA USA; Department of Oral Medicine, Institute of Biomedical Sciences, Tokushima University Graduate School, Tokushima, Japan; Department of Orthodontics, Faculty of Dentistry, King Abdulaziz University, Jeddah, Saudi Arabia

## Abstract

**Introduction:**

Low-intensity pulsed ultrasound (LIPUS) has been known to promote bone healing by nonthermal effects. In recent studies, LIPUS has been shown to reduce inflammation in injured soft tissues. Xerostomia is one of the most common symptoms in Sjögren syndrome (SS). It is caused by a decrease in the quantity or quality of saliva. The successful treatment of xerostomia is still difficult to achieve and often unsatisfactory. The aim of this study is to clarify the therapeutic effects of LIPUS on xerostomia in SS.

**Methods:**

Human salivary gland acinar (NS-SV-AC) and ductal (NS-SV-DC) cells were cultured with or without tumor necrosis factor-α (TNF-α; 10 ng/ml) before LIPUS or sham exposure. The pulsed ultrasound signal was transmitted at a frequency of 1.5 MHz or 3 MHz with a spatial average intensity of 30 mW/cm^2^ and a pulse rate of 20 %. Cell number, net fluid secretion rate, and expression of aquaporin 5 (AQP5) and TNF-α were subsequently analyzed. Inhibitory effects of LIPUS on the nuclear factor κB (NF-κB) pathway were determined by Western blot analysis. The effectiveness of LIPUS in recovering salivary secretion was also examined in a MRL/MpJ/*lpr*/*lpr* (MRL/*lpr*) mouse model of SS with autoimmune sialadenitis.

**Results:**

TNF-α stimulation of NS-SV-AC and NS-SV-DC cells resulted in a significant decrease in cell number and net fluid secretion rate (*p* < 0.01), whereas LIPUS treatment abolished them (*p* < 0.05). The expression changes of AQP5 and TNF-α were also inhibited in LIPUS treatment by blocking the NF-κB pathway. Furthermore, we found that mRNA expression of *A20*, a negative feedback regulator, was significantly increased by LIPUS treatment after TNF-α or interleukin 1β stimulation (NS-SV-AC, *p* < 0.01; NS-SV-DC, *p* < 0.05). In vivo LIPUS exposure to MRL/*lpr* mice exhibited a significant increase in both salivary flow and AQP5 expression by reducing inflammation in salivary glands (*p* < 0.01).

**Conclusions:**

These results suggest that LIPUS upregulates expression of AQP5 and inhibits TNF-α production. Thus, LIPUS may restore secretion by inflamed salivary glands. It may synergistically activate negative feedback of NF-κB signaling in response to inflammatory stimulation. Collectively, LIPUS might be a new strategic therapy for xerostomia in autoimmune sialadenitis with SS.

**Electronic supplementary material:**

The online version of this article (doi:10.1186/s13075-015-0798-8) contains supplementary material, which is available to authorized users.

## Introduction

Sjögren syndrome (SS) is one of the most common chronic autoimmune diseases. It is characterized by lymphocytic infiltrates and destruction of salivary and lacrimal glands [1, 2]. Patients with SS manifest progressive dryness of the mouth and eyes due to insufficient salivary and lacrimal secretions [3].

Currently, the cause of dry mouth (xerostomia) experienced by patients with SS remains unknown. However, dynamic expression of various cytokines has been detected in the salivary glands of humans, as well as in experimental animals, during the development of SS [4, 5]. In particular, expression of tumor necrosis factor (TNF)-α has been strongly associated with decreased salivary flow in patients with SS [6]. It is indicated that TNF-α inhibits salivary secretion due to its neurotoxic effect on sympathetic nerves. Aquaporin 5 (AQP5), a water channel protein, facilitates the rapid transcellular movement of water in response to osmotic and/or hydrostatic pressure gradients [7]. On the basis of its reduced expression and abnormal distribution in the salivary and lacrimal glands of patients with SS, a potential role of AQP5 is proposed.

The treatment of SS remains a challenge because most of the randomized controlled trials done to date have failed to demonstrate efficacy of the treatments evaluated. Hydroxychloroquine (HCQ), one of the most frequently proposed treatments for SS, is currently used on the basis of data obtained from an observational study [8] and a crossover study [9]. However, in a more recent randomized controlled study, the primary endpoint was not achieved [10]. In a recent clinical report regarding the chimeric anti-TNF-α antibody infliximab, patients with SS were found to exhibit a dramatic improvement in salivary flow [11], though there have been a few scattered reports of anti-TNF-α treatment inefficacy [12, 13]. Furthermore, Yamamura et al. [3] found that TNF-α stimulation dramatically decreased water flow rate (e.g., salivary flow) in cultured acinar cells of human salivary glands, supporting the effectiveness of infliximab. Thus, accumulating evidence suggests that anticytokine therapy that includes targeted inhibition of TNF-α activity may represent a treatment for xerostomia caused by autoimmune sialadenitis in SS. However, a significant number of patients taking HCQ may be at an increased risk for retinal toxicity [14], and anti-TNF-α treatment has many side effects, including anaphylaxis [15], and increased risk for infections, such as tuberculosis and demyelination, aplastic anemia, intestinal perforation, lymphoma, and congestive heart failure [16]. In other words, the establishment of a treatment with a better side effect profile is urgently needed.

Low-intensity pulsed ultrasound (LIPUS) has been used extensively as a therapeutic, operative, and diagnostic tool in medicine. Previous studies have demonstrated that LIPUS can promote bone repair and regeneration, accelerate bone fracture healing [17, 18], and enhance osteogenesis at the distraction site [19, 20]. Therefore, LIPUS is well-accepted as a noninvasive and safe therapeutic tool for the treatment of bone fractures [21].

Recently, the effect of ultrasound on soft tissues has received much attention. It has been reported that LIPUS promotes cell proliferation and the synthesis of extracellular matrix in fibroblasts and myoblasts [22–24]. Furthermore, ultrasound has been shown to reduce inflammation and promote regeneration in various injured soft tissues [23, 25, 26]. Thus, ultrasound therapy may have considerable clinical potential for shortening the healing time of injured or pathological soft tissues. However, little information is available regarding the effect of LIPUS on salivary glands and the acinar and ductal cells that compose them. Accordingly, the aim of this study was to examine the inhibitory effects of LIPUS on cell proliferation, net fluid secretion rate, and AQP5 expression of TNF-α-stimulated normal human salivary gland acinar and ductal cells in vitro. In addition, we analyzed the effect of LIPUS on the nuclear factor κB (NF-κB) signaling pathway in the acinar and ductal cells stimulated with TNF-α. We also evaluated improvement of the effects of LIPUS on xerostomia in a mouse model of SS.

## Methods

### Cell culture

Immortalized clones of human salivary gland acinar (NS-SV-AC) and ductal (NS-SV-DC) cells have been generated [27]. These cells were cultured at 37 °C in serum-free keratinocyte medium (SFKM; Gibco/Thermo Fisher Scientific, Grand Island, NY, USA) in an atmosphere containing 5 % CO_2_.

### Animals

A total of 10 C57BL/6 and 20 MRL/MpJ/*lpr*/*lpr* (MRL/*lpr*) female mice were purchased from Japan SLC (Hamamatsu, Japan) and were used as the control and experimental groups, respectively. Mice were kept at a constant ambient temperature (22–24 °C) with a 12-h/12-h light/dark cycle and received a solid diet ad libitum in the animal facility of Tokushima University under specific pathogen-free conditions. The experimental protocol described below was approved by the ethics committee of Tokushima University (permit number 13015). The control groups included 6-week-old, 12-week-old, 20-week-old, and 24-week-old mice (n = 5 for each). The experimental groups included 12-week-old and 20-week-old mice (n = 5 for each).

### LIPUS

LIPUS was applied by a modified version of the ST-Sonic clinical device (ITO Co., Tokyo, Japan). The modified system consisted of a 5.0-cm^2^ circular surface transducer and a cell culture plate. The ultrasound head had a mean beam nonuniformity value of 2.7 and an effective radiating area of 4.1 cm^2^. An ultrasound signal was transmitted at a frequency of 3 MHz in vitro and 1.5 MHz in vivo. It had a spatial average intensity value of 30 mW/cm^2^ and a pulse rate of 1:4 (2 ms on and 8 ms off). A six-well plate was maintained in vitro with its top above water level in a foam-fronted plastic sliding assembly containing an aperture of dimensions matching to the monolayer (Additional file 1: Figure S1). The distance between the transducer and the cells was approximately 1 mm. The cell cultures were treated with 20 min of a single ultrasound exposure. The tank water was maintained at 37 ± 0.5 °C. The LIPUS exposure assembly was maintained in a humidified atmosphere of 5 % CO_2_ at 37 °C during all experiments, and an electronic control panel activated an alert signal if the coupling gel or liquid was depleted. Control samples were treated in parallel, although LIPUS was not applied. The in vivo submandibular glands of the mice received 20 min of LIPUS per day for 14 days.

### Cell proliferation

Cells were grown in 96-well microplates (2 × 10^4^ cells/well) in SFKM. After the appropriate incubation period, the number of attached cells was counted using a Z1 COULTER COUNTER (Beckman Coulter, Fullerton, CA, USA). Moreover, the 2-(2-meth-oxy-4-nitrophenyl)-3-(4-nitrophenyl)-5-(2,4-disulfophenyl)-2H-tetrazolium monosodium salt assay was performed using Cell Count Reagent SF (Nacalai Tesque, Kyoto, Japan). The results were obtained at day 0 as a baseline reading. Subsequently, the culture medium was removed and the cells were stimulated with or without TNF-α (10 ng/ml). Four hours later, the cultured cells were exposed to LIPUS or sham exposure. After 1 day of culturing, the cell numbers were counted again. Cell proliferation was evaluated based on the ratio of treated cells to untreated control cells.

### Immunofluorescence

Cells grown on a coverslip were treated with or without TNF-α (10 ng/ml) for 4 h, then received LIPUS or sham exposure. Twenty-four hours later, the cells were washed twice with phosphate-buffered saline (PBS), fixed in 4 % paraformaldehyde in PBS for 20 min, and then incubated for 1 h at 37 °C with goat antihuman AQP5 antibody (1:100, sc-28628; Santa Cruz Biotechnology, Santa Cruz, CA, USA). After three washes with PBS containing 1 % bovine serum albumin, the cells were incubated for 1 h with Alexa Flour 488–conjugated secondary antibody (1:1000; Cell Signaling Technology, Danvers, MA, USA) at room temperature (RT) in the dark. After the unbound antibodies were washed away, coverslips were mounted using fluorescence mounting medium (Dako, Glostrup, Denmark). Bound antibody was observed using a fluorescence microscope (BZ-9000; KEYENCE, Osaka, Japan), and the fluorescence intensity was quantified by using the BZ analyzer (KEYENCE).

### Net fluid secretion rate measurements

The net fluid secretion rates for NS-SV-AC and NS-SV-DC cells treated with or without TNF-α (10 ng/ml), as well as those that additionally received LIPUS or sham exposure, were measured using a modified method described previously [28]. Briefly, 4 h after LIPUS or sham exposure, the liquid on the apical side was collected and its volume was measured using a calibrated pipette.

### RNA isolation and real-time polymerase chain reaction analysis

Cultured NS-SV-AC and NS-SV-DC cells were treated with or without TNF-α (10 ng/ml) or interleukin (IL)-1β (1 ng/ml) and received LIPUS or sham exposure. After 4 h, total cellular RNA was extracted using NucleoSpin RNA (MACHEREY-NAGEL, Düren, Germany). First-strand cDNA was synthesized from total RNA (1000 ng) using a High Capacity RNA-to-cDNA Kit (Applied Biosystems, Foster City, CA, USA). Using real-time PCR analysis with StepOnePlus (Applied Biosystems) and TaqMan Fast Advanced Master Mix (Applied Biosystems), mRNA levels of *AQP5*, *TNF-α*, and *A20* were examined. The following TaqMan probe mixtures were used: TaqMan gene expression assays; *AQP5*, Hs00387048_m1; *TNF-α*, Hs01113624_g1; *A20*, Hs00234713_m1; and *β-actin*, Hs01060665_g1 (Applied Biosystems). The cycling conditions included 20 s at 95 °C, 40 cycles of 1 s at 95 °C, and 20 s at 60 °C. Detection of β-actin was used as an internal control. Expression of *AQP5*, *TNF-α*, and *A20* were calculated using the cycle threshold method.

### Western blot analysis

Cultured in vitro NS-SV-AC and NS-SV-DC cells were treated with or without TNF-α (10 ng/ml) or IL-1β (1 ng/ml) and then received LIPUS or sham exposure. After 24 h, the cells were precipitated and lysed with M-PER Mammalian Protein Extraction Reagent (Thermo Fisher Scientific, Waltham, MA, USA). Salivary glands were homogenized in vivo with T-PER Mammalian Protein Extraction Reagent (Thermo Fisher Scientific). The samples were centrifuged, and the protein concentration of each supernatant was measured using a bicinchoninic acid protein assay kit (Thermo Fisher Scientific) and microplate reader (Corona Electric, Hitachinaka, Japan). SDS-PAGE was used to separate each 20-μg sample in vitro and each 40-μg sample in vivo, and the separated proteins were then transferred electrophoretically onto polyvinylidene difluoride membranes (EMD Millipore, Billerica, MA, USA). The membranes were blocked for 1 h at RT with 0.1 % Tris-buffered saline with Tween 20 (TBS-T) containing 3 % skim milk, then incubated overnight at 4 °C with antihuman AQP5 antibody (1:500; Santa Cruz Biotechnology), anti-TNF-α antibody (1:500, catalog number 3707; Cell Signaling Technology), phosphorylated inhibitor of nuclear factor of κ light polypeptide gene enhancer in B cells, α subunit (phospho-IκBα) antibody (1:1000, catalog number 9246; Cell Signaling Technology), IκBα antibody (1:1000, catalog number 9242; Cell Signaling Technology), phospho-NF-κB p65 antibody (1:1000, catalog number 3033; Cell Signaling Technology), NF-κB p65 antibody (1:1000, catalog number 8242; Cell Signaling Technology), phosphorylated inhibitor of nuclear factor κB kinase subunit β (phospho-IKKβ) antibody (1:1000, catalog number 2697; Cell Signaling Technology), IKKβ antibody (1:1000, catalog number 2678; Cell Signaling Technology), interleukin 1 receptor-associated kinase 1 (IRAK1) antibody (1:1000, catalog number 4504; Cell Signaling Technology), or anti-β-actin antibody (1:1000, catalog number 4967; Cell Signaling Technology) in TBS-T. The membranes were washed three times with TBS-T for 15 min and then incubated for 1 h with the appropriate secondary antibodies conjugated to horseradish peroxidase (HRP). Bound antibodies were visualized using a Western blot detection system with LumiGLO reagent (Cell Signaling Technology) according to the manufacturer’s instructions. Protein bands were quantitated in vivo by densitometric analysis using image analysis software (CS Analyzer; ATTO, Tokyo, Japan).

### Fluid secretion measurements

Control mice (aged 6, 12, 20, and 24 weeks old) and experimental mice (12 and 20 weeks old) had their salivary volumes measured following LIPUS treatment. A modified measurement method described previously was used [29]. Briefly, an intramuscular injection of pilocarpine (5 mg/kg) was administered without anesthesia. The total volume of saliva was then determined gravimetrically after a 20-minute collection period according to a method used in a Saxon test for the diagnosis of patients with SS [30].

### Histology

After measuring fluid secretion, all salivary glands were resected, fixed with 4 % phosphate-buffered formaldehyde (pH 7.2), and prepared for histological examination. Formalin-fixed tissue sections (6 μm) were then subjected to hematoxylin and eosin staining, and three pathologists independently evaluated the histology without being informed of the condition of each mouse.

Histological grading was performed according to a previously proposed method [31]. Briefly, longitudinal sections of all glands were examined at × 150 magnification and scored for the degree of inflammatory infiltrate observed. Scoring ranged from 1 to 4 and was used to indicate that 1= 1–5 foci of mononuclear cells were observed among more than 20 cells; 2= more than 5 such foci were observed without significant parenchymal destruction; 3= multiple confluent loci were observed with moderate degeneration of parenchymal tissue; and 4= extensive infiltration of the glands with mononuclear cells and extensive parenchymal destruction were observed, respectively.

### Immunohistochemical staining of AQP5

Sections of salivary glands were deparaffinized and rehydrated in a xylene-ethanol series. After the endogenous peroxidases in each section were blocked, the sections were incubated overnight with an antihuman AQP5 antibody (1:200 in an antibody solution buffer; Santa Cruz Biotechnology) at 4 °C. After the sections were washed three times with PBS, they were incubated with EnVision + Rabbit/HRP (Dako) as a secondary antibody. Immunoreactivity was detected using diaminobenzidine (Dako), and each section was counterstained with Mayer’s hematoxylin.

### Statistical analysis

Mean and standard deviation values were calculated. Significant differences in experimental data were analyzed by one-way analysis of variance, followed by the Tukey–Kramer test and the Bonferroni–Dunn test as a post hoc test to examine mean differences at the 5 % level of significance.

## Results

### Effects of LIPUS on cell proliferation

**Fig. 1:**
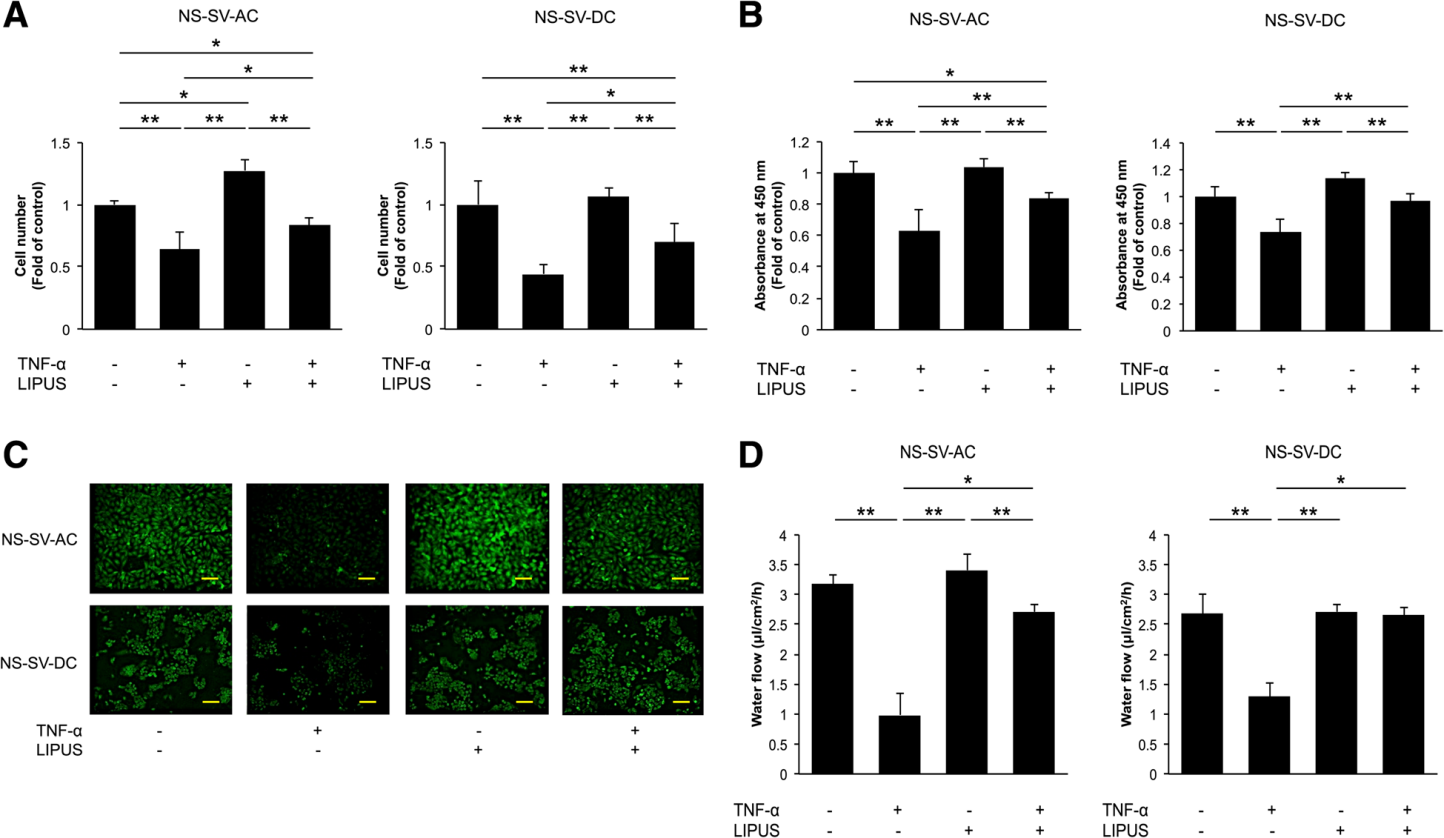
Effects of LIPUS on NS-SV-AC and NS-SV-DC cells. **a** Following TNF-α stimulation, a significant decrease in cell numbers was observed for both cell types. These numbers increased following a single exposure to LIPUS. These data are presented as the fold number relative to each control cell type. *p < 0.05; **p < 0.01 (n = 6). **b** 2-(2-methoxy-4-nitrophenyl)-3-(4-nitrophenyl)-5-(2,4-disulfophenyl)-2H-tetrazolium monosodium salt assay showed results similar to the cell count assay for both cell types. *p < 0.05; **p < 0.01 (n = 5). **c** Immunofluorescence assays were performed to detect expression of AQP5 (*green*) in NS-SV-AC and NS-SV-DC cells. Staining intensity markedly decreased following TNF-α treatment, whereas TNF-α plus LIPUS restored AQP5 expression to levels exhibited by untreated cells. Scale bar = 100 μm. **d** The net water flow (e.g., fluid secretion rates) for NS-SV-AC and NS-SV-DC cells were significantly lower following TNF-α stimulation, whereas TNF-α plus LIPUS significantly increased the rate for both cell types. *p < 0.05; **p < 0.01 (n = 3). *AQP5* aquaporin 5, *LIPUS* low-intensity pulsed ultrasound, *NS-SV-AC* salivary gland acinar cells, *NS-SV-DC* salivary gland ductal cells, *TNF-α* tumor necrosis factor α

Compared with untreated control cells, cells stimulated with TNF-α showed a significant decrease in the number and proliferation of adherent NS-SV-AC and NS-SV-DC cells that were detected (*p* < 0.01) (Fig. 1a, b). In contrast, the subsequent LIPUS treatment induced a significant increase in proliferation for both NS-SV-AC and NS-SV-DC cells stimulated with TNF-α (*p* < 0.01). Treatment with LIPUS alone had no catabolic effect on cell proliferation.

### AQP5 expression in NS-SV-AC and NS-SV-DC cells

Untreated NS-SV-AC cells exhibited intense AQP5 expression compared with untreated NS-SV-DC cells (Fig. 1c, Additional file 2: Figure S2). However, following treatment with TNF-α, AQP5 expression markedly decreased, especially in the NS-SV-AC cells. When NS-SV-AC and NS-SV-DC cells were treated with TNF-α and then LIPUS, AQP5 levels increased to match the baseline level of AQP5 expression exhibited by untreated cells. In contrast, LIPUS treatment alone had no effect on AQP5 expression.

### Fluid secretion rate in NS-SV-AC and NS-SV-DC cells

Compared with the secretion rate of untreated control cells, stimulation with TNF-α resulted in a significant decrease in the net fluid secretion rate of NS-SV-AC and NS-SV-DC cells (*p* < 0.01) (Fig. 1d). However, when LIPUS treatment was administered following TNF-α treatment, significant increases in the net fluid secretion rate of both cells were observed (*p* < 0.05).

### Gene and protein expression of AQP5 and TNF-α in NS-SV-AC and NS-SV-DC cells treated with TNF-α

**Fig. 2:**
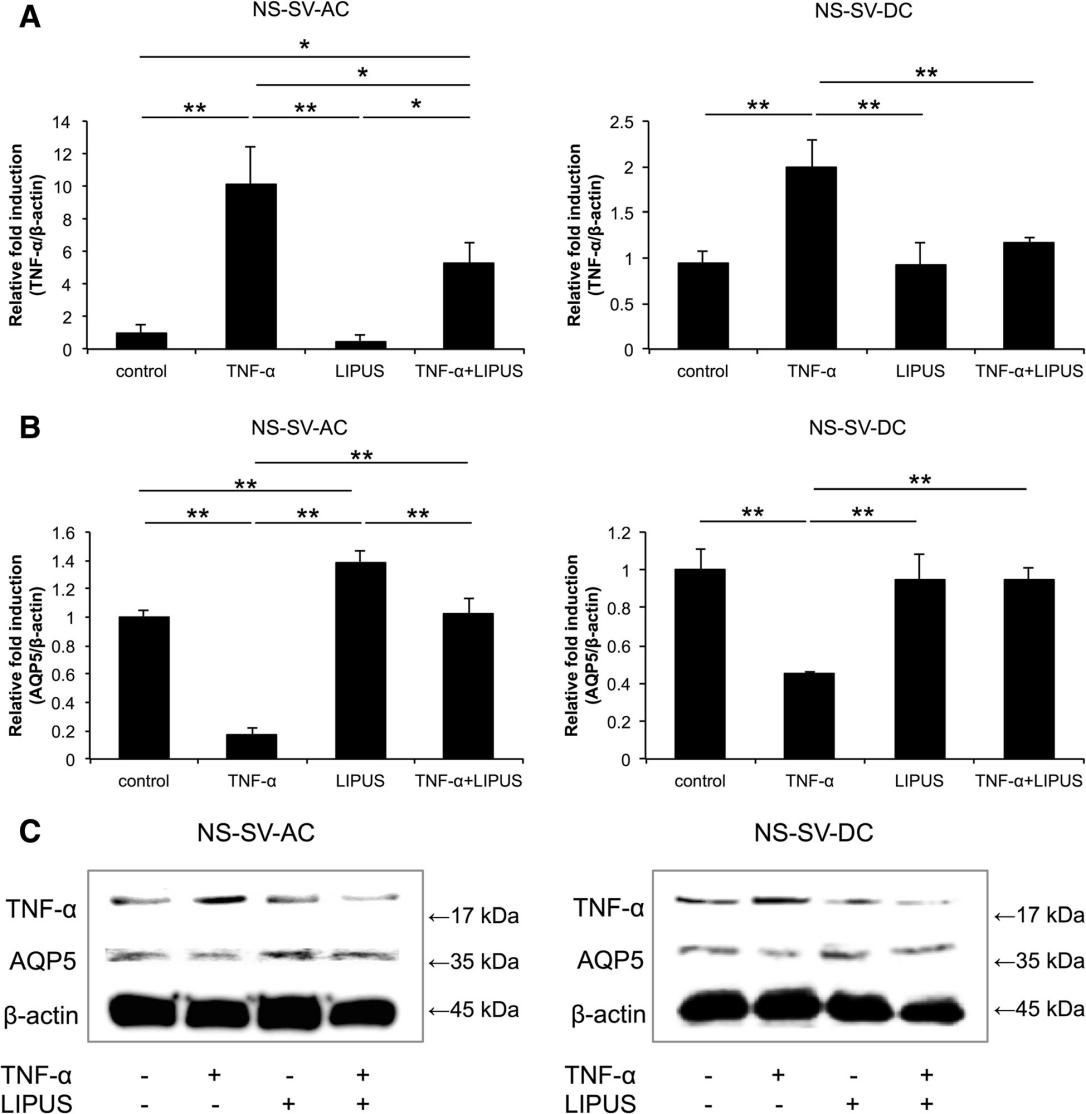
Gene and protein expression of TNF-α and AQP5 in NS-SV-AC and NS-SV-DC cells. TNF-α stimulation of NS-SV-AC and NS-SV-DC cells significantly increased levels of *TNF-α* mRNA (**a**) and decreased levels of *AQP5* mRNA. (**b**). Detection of *β-actin* mRNA was used to calculate relative fold induction for each. However, when TNF-α stimulation was followed by LIPUS, both levels were restored to those of control cells. *p < 0.05; **p < 0.01 (n = 6). **c** In Western blot assays, the expression of the AQP5 was decreased following TNF-α stimulation, whereas the expression of TNF-α was increased, in both cell types. When LIPUS was performed following TNF-α treatment, levels of AQP5 were increased in both cell types compared with untreated controls. *AQP5* aquaporin 5, *LIPUS* low-intensity pulsed ultrasound, *NS-SV-AC* salivary gland acinar cells, *NS-SV-DC* salivary gland ductal cells, *TNF-α* tumor necrosis factor α

Compared with control cells, the stimulation of both NS-SV-AC and NS-SV-DC cells with TNF-α resulted in a significant decrease in levels of *AQP5* mRNA (*p* < 0.01) and a significant increase in levels of *TNF-α* mRNA (*p* < 0.01) (Fig. 2a, b). However, the latter was reversed following treatment with LIPUS (NS-SV-AC, *p* < 0.05; NS-SV-DC, *p* < 0.01). Moreover, LIPUS treatment of untreated NS-SV-AC cells resulted in a significant increase in levels of *AQP5* mRNA (Fig. 2a, b).

The intensity of the AQP5 band detected was decreased following TNF-α stimulation, whereas expression of TNF-α was clearly enhanced (Fig. 2c, Additional file 3: Figure S3). However, when LIPUS treatment was performed after TNF-α treatment, an increase in AQP5 levels was observed in both NS-SV-AC and NS-SV-DC cell extracts compared with untreated control cells. In contrast, AQP5 levels of untreated cells that received LIPUS treatment alone exhibited minimal, if any, change in signal intensity.

### Mechanism of LIPUS on NS-SV-AC and NS-SV-DC cellular function

**Fig. 3:**
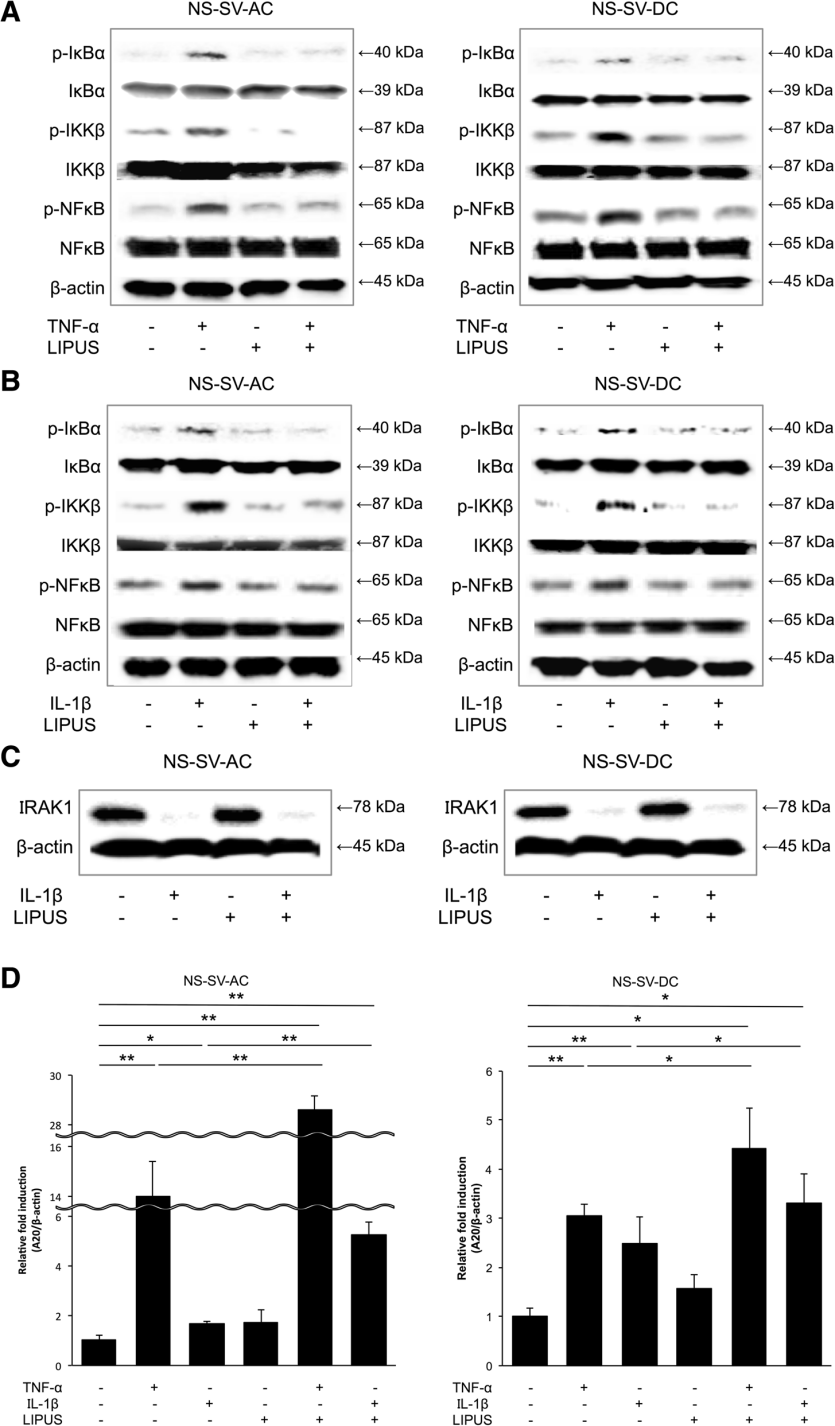
Anti-inflammatory mechanism of LIPUS on NS-SV-AC and NS-SV-DC cellular function. **a** Western blot analysis showed IκBα phosphorylation was obviously induced by TNF-α stimulation; however, when LIPUS treatment was administered following TNF-α treatment, IκBα, NF-κB, and IKKβ phosphorylation were inhibited. **b** Similar inhibitory effects of LIPUS were observed in IL-1β stimulation. **c** In Western blot analysis, IRAK1 was degraded after IL-1β stimulation, and LIPUS exposure failed to inhibit it. **d** The stimulation of both NS-SV-AC and NS-SV-DC cells with TNF-α or IL-1β resulted in a significant increase in levels of *A20* mRNA, and *A20* mRNA expression was further increased following treatment with LIPUS after TNF-α or IL-1β stimulation. *p < 0.05; **p < 0.01 (n = 6). *A20* tumor necrosis factor-α-induced protein 3 (*TNFAIP3*), *IKKβ* inhibitor of nuclear factor κB kinase subunit β, *IL-1β* interleukin 1β, *IRAK1* interleukin 1 receptor-associated kinase 1, *IκBα* inhibitor of nuclear factor of κ light polypeptide gene enhancer in B cells, α subunit, *LIPUS* low-intensity pulsed ultrasound, *NF-κB* nuclear factor κB, *NS-SV-AC* salivary gland acinar cells, *NS-SV-DC* salivary gland ductal cells, *TNF-α* tumor necrosis factor α

To determine whether LIPUS inhibits inflammation in NS-SV-AC and NS-SV-DC cells through the prevention of NF-κB activation, we investigated the effect of IκBα, NF-κB, and IKKβ phosphorylation inhibition on phosphorylation of proteins involved in anti-inflammatory effects. Western blot analysis showed IκBα, NF-κB, and IKKβ phosphorylation were obviously induced by TNF-α stimulation; however, LIPUS exposure following TNF-α treatment inhibited IκBα, NF-κB, and IKKβ phosphorylation in both NS-SV-AC and NS-SV-DC cells (Fig. 3a, Additional file 4: Figure S4A). In addition, IκBα, NF-κB, and IKKβ phosphorylation induced by IL-1β stimulation was also inhibited by LIPUS exposure (Fig. 3b, Additional file 4: Figure S4B). This indicates that anti-inflammatory effects of LIPUS are not a specific response to TNF-α stimulation.

Furthermore, to determine LIPUS effects on the upstream of NF-κB signaling pathway, we examined IRAK1 expression on IL-1β stimulation. IRAK1 was degraded by IL-1β stimulation, and LIPUS exposure failed to inhibit the degradation of IRAK1 induced by IL-1β stimulation (Fig. 3c, Additional file 4: Figure S4C). This means that the effect point of LIPUS on the NF-κB signaling pathway is downstream of IRAK1. Next, we examined *A20* (tumor necrosis factor-α-induced protein 3 [*TNFAIP3*]) mRNA expression using real-time PCR. A20 is known as the intracellular ubiquitin-editing protein and a key player in the negative feedback of NF-κB signaling in response to multiple stimulation [32]. Compared with untreated control cells, the stimulation of both NS-SV-AC and NS-SV-DC cells with TNF-α or IL-1β resulted in a significant increase in levels of *A20* mRNA (*p* < 0.01). Furthermore, *A20* mRNA expression was more increased by treatment with LIPUS after TNF-α or IL-1β stimulation (NS-SV-AC, *p* < 0.01; NS-SV-DC, *p* < 0.05) (Fig. 3d). These data show that LIPUS activates A20, which produces negative feedback of NF-κB signaling in response to inflammatory stimulation, resulting in inhibition of inflammation in salivary gland cells.

### Changes in fluid secretion volumes with aging

**Fig. 4:**
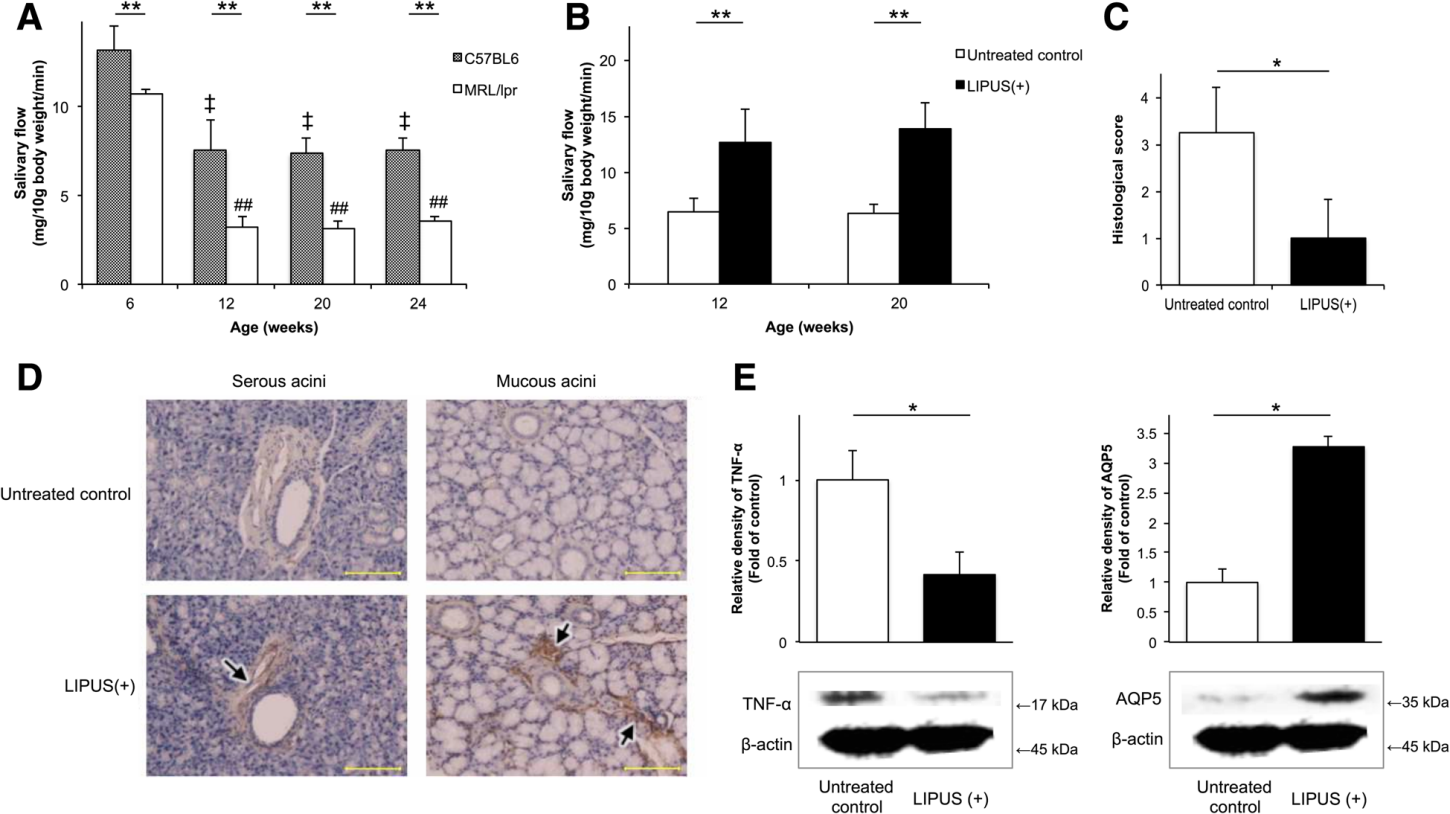
In vivo effects of LIPUS on MRL/*lpr* mice. **a** The average salivary volumes of 12-week-old, 20-week-old, and 24-week-old MRL/*lpr* mice were lower those of 6-week-old MRL/*lpr* mice. The average salivary secretion of the MRL/*lpr* mice at each age was also significantly lower than that for C57BL/6 mice at the same ages. **p < 0.01; ^‡^ *p* < 0.01 compared with 6-week-old C57BL/6 mice; ^##^ *p* < 0.01 compared with 6-week-old MRL/*lpr* mice (n = 5). **b** Both 12-week-old and 20-week-old MRL/*lpr* mice showed a significant increase in salivary flow following LIPUS treatment. **p < 0.01 (n = 5). **c** Histological scores for inflammatory lesions present in the salivary glands of untreated versus LIPUS-treated MRL/*lpr* mice. LIPUS treatment significantly improved the histological score. *p < 0.05 (n = 5). **d** In both the serous and mucous acini of the submandibular glands, acinar cells of untreated MRL/*lpr* mice exhibited weak staining for AQP5, whereas intense staining of AQP5 was localized to apical sites in acinar cells of MRL/*lpr* mice treated with LIPUS (*arrows*). Scale bar = 100 μm. **e** In Western blot assays, levels of TNF-α were significantly lower, and levels of AQP5 were significantly higher, following LIPUS treatment compared with untreated MRL/*lpr* samples. Detection of β-actin was used to calculate relative fold induction for each. *p < 0.05 (n = 5). *AQP5* aquaporin 5, *LIPUS* low-intensity pulsed ultrasound, MRL/*lpr* MRL/MpJ/*lpr*/*lpr*, *TNF-α* tumor necrosis factor α

The average salivary volumes for 12-week-old, 20-week-old, and 24-week-old MRL/*lpr* mice were significantly lower than those obtained from 6-week-old mice (*p* < 0.01) (Fig. 4a). Moreover, compared with C57BL/6 mice, the average salivary secretion volumes for the MRL/*lpr* mice were significantly lower at all ages (*p* < 0.01).

### In vivo effect of LIPUS on salivary secretion in MRL/*lpr* mice

To examine the effect of LIPUS on salivary secretion in MRL/*lpr* mice, secreted saliva volumes were measured using a modified method employed in the Saxon test [21]. Following LIPUS treatment, salivary secretion was found to be restored in both 12-week-old and 20-week-old MRL/*lpr* mice to a degree comparable to that of younger C57BL/6 mice (Fig. 4b). Furthermore, both 12-week-old and 20-week-old MRL/*lpr* mice exhibited a significant increase in salivary secretion following LIPUS treatment compared with untreated MRL/*lpr* mice.

### Anti-inflammatory effect of LIPUS on salivary glands in MRL/*lpr* mice

MRL/*lpr* mice treated with LIPUS showed a marked reduction in histological damage, such as the lymphocyte infiltration of surrounding duct and the destruction of gland tissue, compared with untreated MRL/*lpr* mice (Additional file 5: Figure S5A). Moreover, the histological score for the inflammatory lesions of the submandibular glands of MRL/*lpr* mice was significantly improved following LIPUS (*p* < 0.05) (Fig. 4c).

### AQP5 expression in salivary glands

As shown in Fig. 4d, submandibular gland acinar cells of untreated MRL/*lpr* mice showed weak staining for AQP5 expression (brown stain) in both the serous and mucous acini of the submandibular glands. In contrast, strong expression of AQP5 was observed at apical sites in submandibular gland acinar cells of MRL/*lpr* mice treated with LIPUS.

### Induction of AQP5 and TNF-α expression in salivary glands

Following LIPUS exposure, significantly lower levels of TNF-α (*p* < 0.05) and significantly higher levels of AQP5 (*p* < 0.05) were detected in the salivary glands of treated MRL/*lpr* mice compared with untreated MRL/*lpr* mice (Fig. 4e, Additional file 5: Figure S5B).

## Discussion

To our knowledge, the effect of LIPUS on salivary gland cells has not previously been investigated. Thus, the present study would be the first to investigate the effect of LIPUS on cell proliferation and the synthesis of AQP5 in normal human salivary gland acinar and ductal cells stimulated with TNF-α. The present results indicate that LIPUS is able to upregulate cell proliferation and AQP5 expression in both of these stimulated cell types. Furthermore, using a mouse model of autoimmune sialadenitis in SS, LIPUS treatment was found to improve secretion from salivary glands in both age-related and SS xerostomia experiments.

Although the mechanisms mediating LIPUS-stimulated tissue repair have not yet been elucidated, it is hypothesized that the anabolic biophysical effects of ultrasound are due to mechanical stress and/or the impact of fluid microstreaming on the cellular plasma membrane, focal adhesions, and cytoskeletal structures. As a result, intracellular signal transduction is activated and gene transcription is affected [33]. There is evidence to suggest that LIPUS activates integrins on the cell surface that act as mechanoreceptors to promote the attachment of various focal adhesion adaptor proteins [34]. In addition, levels of phosphorylated focal adhesion kinase (FAK) in synovial cells were found to increase following LIPUS treatment [35, 36]. Furthermore, LIPUS treatment of cementoblasts has been shown to enhance expression of extracellular signal-regulated kinase 1/2 [35]. Because the proliferation of various cell types is mediated by growth factors or cytokine-induced mitogen-activated protein kinase (MAPK) proteins [37], the exposure of salivary gland cells to LIPUS may lead to specific activation of an integrin/FAK/MAPK pathway.

In human salivary glands, AQP5 localizes to the apical membranes of acinar cells and stimulates the outflow of water into the acinar lumen [38]. In patients with SS, the salivary and lacrimal glands exhibit a marked reduction in AQP expression in the plasma membrane [39], as well as a delocalization of AQP5 to the basal membrane [40]. Correspondingly, reduced salivary gland secretion has been observed in mice harboring a mutant AQP5 channel [41]. Therefore, although the molecular mechanisms by which AQP5 dysfunction is induced in the salivary and lacrimal glands of patients with SS remains unknown, it appears that enhancement of AQP5 expression in salivary acinar cells may be a critical aspect. In the present study, expression of AQP5 in NS-SV-AC and NS-SV-DC cells was inhibited by TNF-α treatment, whereas it was recovered following LIPUS exposure. Furthermore, AQP5 synthesis and expression in the salivary glands of MRL/*lpr* mice was restored following LIPUS treatment, resulting in the restoration of salivary flow to normal levels.

In this study, we hypothesize that this event is due to the anti-inflammatory effects mediated by LIPUS treatment through the prevention of NF-κB activation. NF-κB is composed of homo- and heterodimeric complexes of members of the Rel protein family. NF-κB normally resides in the cytoplasm, where it is retained by association with IκB protein, an endogenous inhibitor. Various extracellular stimuli trigger the degradation of IκB by the proteasome pathway. Subsequently, NF-κB released from IκB translocates into the nucleus, binds to the regulatory element of the target genes, and controls their transcription [42]. Our results demonstrated that the anti-inflammatory effects of LIPUS in NS-SV-AC and NS-SV-DC cells were involved in the inhibition of NF-κB signaling pathway. Interestingly, LIPUS activated the intracellular ubiquitin-editing protein A20 in inflammatory stimulation. A20, a cytoplasmic zinc finger protein, was originally identified as a TNF-inducible protein. It has been characterized as a dual inhibitor of NF-κB activation and cell death [31] and functions as a negative feedback regulator of NF-κB activation via multiple mechanisms [43]. Furthermore, A20 inhibits TNF- and IL-1-induced NF-κB activation in 293 cells [44], and deregulated Toll-like receptor signaling in response to commensal bacteria was shown to be responsible for the multiorgan inflammation and premature death of A20-knockout mice [45]. Thus, LIPUS may inhibit the NF-κB pathway by activating a negative feedback system in the salivary gland acinar and ductal cells.

In IL-1β-stimulated synovial membrane cells, increased expression of cyclooxygenase 2 (*Cox-2*), a NF-κB-responsible gene, was found to be significantly inhibited by LIPUS in vitro [24]. Expression of *Cox-2* in the knee joints of MRL/*lpr* mice was also found to be markedly reduced following daily treatments with LIPUS [25], whereas inhibition of *Cox-2* was found to reduce the proliferation and induce the apoptosis of human cholangiocarcinoma QBC939 cells via inhibition of prostaglandin E_2_ production [46]. On the basis of these data, it is hypothesized that inhibition of *Cox-2* expression by LIPUS restores salivary secretion in MRL/*lpr* mice as a secondary effect.

It is generally accepted that salivary secretion volume decreases with age. The prevalence of xerostomia also increases with age and affects approximately 30 % of people aged 65 years or older [47]. In contrast, the production and composition of saliva remains largely independent of age in healthy individuals [48, 49]. Recently, Yamamura et al. [50] postulated that age-related hypermethylation of the *AQP5* gene could account for the downregulation of AQP5 expression that is observed in the salivary glands of patients with SS. Correspondingly, demethylation of the *AQP5* promoter in salivary gland cells by 5-aza-2′-deoxycytidine (decitabine) could potentially restore salivary flow in aged mice. The results of the present study demonstrate that the average salivary volumes of the 12-week-old and older MRL/*lpr* and wild-type mice were significantly lower than those of the 6-week-old mice. In addition, LIPUS treatment restored the salivary secretion volume of both the 12-week-old and 20-week-old MRL/*lpr* mice to the salivary flow level observed in the younger wild-type mice. Thus, it appears that reduced salivary flow in older MRL/*lpr* mice is a consequence of autoimmune disease as well as the aging process, and LIPUS treatment can compensate for these processes. However, further studies are needed to identify the effect of LIPUS on age-related xerostomia to understand how LIPUS affects salivary glands and their cells.

In recent years, LIPUS has been paid attention as a physical therapy that has insignificant side effects. In this study, we investigated the effectiveness of LIPUS on xerostomia associated with SS, and the results suggest that LIPUS might rescue salivary secretion volume in an SS mouse model by their anti-inflammatory effect in the salivary gland tissue. Furthermore, LIPUS has much potential for clinical application because it can be used in combination with conventional pharmacotherapy. Our results also suggest that further studies to determine clinical efficacy, safety, and response duration are warranted.

## Conclusions

LIPUS treatment was found to increase cell proliferation and AQP5 expression in salivary gland cells pretreated with TNF-α in vitro. Moreover, LIPUS activates the intracellular ubiquitin-editing protein A20, which produces negative feedback of NF-κB signaling in response to inflammatory stimulation. LIPUS exposure also restored salivary gland secretion volumes in older MRL/*lpr* mice in vivo, thereby promoting an anti-inflammatory response and improving AQP5 dysfunction. Therefore, LIPUS stimulation may represent a treatment strategy for inflammatory diseases of salivary glands, including xerostomia in SS.

## Additional files

Additional file 1: Figure S1. Schematic representation of the in vitro LIPUS system used. A cell culture plate with medium was placed in the ultrasound field at a distance of about 1 mm from the transducer to optimize beam uniformity across the target region. (TIFF 1093 kb) Additional file 2: Figure S2. Quantification of the fluorescence intensity in Fig. 1C. ***p* < 0.01 (n = 4). (TIFF 897 kb) Additional file 3: Figure S3. Bands of Western blot analysis for the cropped images in Fig. 2c are provided in this file. (TIFF 1856 kb) Additional file 4: Figure S4. Bands of Western blot analysis for the cropped images in Fig. 3a **(A)**, 3b **(B)** and 3c **(C)** are provided in this file. (TIFF 5449 kb) Additional file 5: Figure S5.
**(A)** MRL/*lpr* mice treated with LIPUS exhibited a marked reduction in histological damage compared with untreated MRL/*lpr* mice. Scale bar = 100 μm. **(B)** Bands of Western blot analysis for the cropped images in Fig. 4e are provided in this file. (TIFF 5901 kb)

## Abbreviations

*A20*: tumor necrosis factor-α-induced protein 3 (*TNFAIP3*)
AQP5: aquaporin 5
Cox-2: cyclooxygenase 2
FAK: focal adhesion kinase
HCQ: hydroxychloroquine
HRP: horseradish peroxidase
IKKβ: inhibitor of nuclear factor κB kinase subunit β
IL-1β: interleukin 1β
IRAK1: interleukin 1 receptor-associated kinase 1
IκBα: inhibitor of nuclear factor of κ light polypeptide gene enhancer in B cells, α subunit
LIPUS: low-intensity pulsed ultrasound
MAPK: mitogen-activated protein kinase
MRL/*lpr*: MRL/MpJ/*lpr*/*lpr*
NF-κB: nuclear factor κB
NS-SV-AC: salivary gland acinar cells
NS-SV-DC: salivary gland ductal cells
PBS: phosphate-buffered saline
RT: room temperature
SFKM: serum-free keratinocyte medium
SS: Sjögren syndrome
TBS-T: Tris-buffered saline with Tween 20
TNF-α: tumor necrosis factor α
