# Supplementary figures and images for: Low-intensity pulsed ultrasound rescues insufficient salivary secretion in autoimmune sialadenitis
Source: Arthritis Res Ther. 2015 Oct 7;17:278. doi: 10.1186/s13075-015-0798-8 (PMC4596462; doi:10.1186/s13075-015-0798-8)

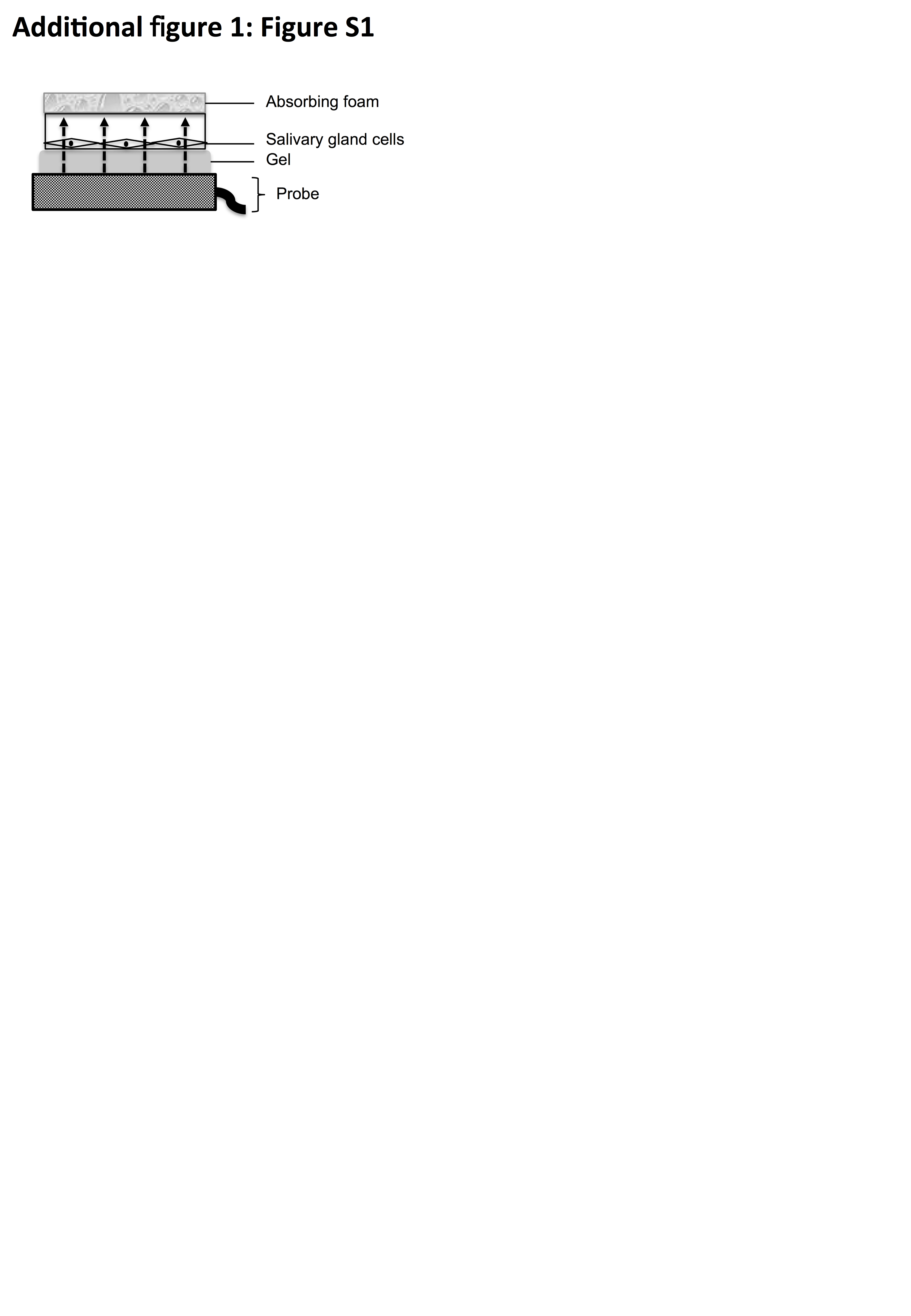

Supplement: Additional file 1: Figure S1. — Schematic representation of the in vitro LIPUS system used. A cell culture plate with medium was placed in the ultrasound field at a distance of about 1 mm from the transducer to optimize beam uniformity across the target region. (TIFF 1093 kb) [file 13075_2015_798_MOESM1_ESM.tiff]

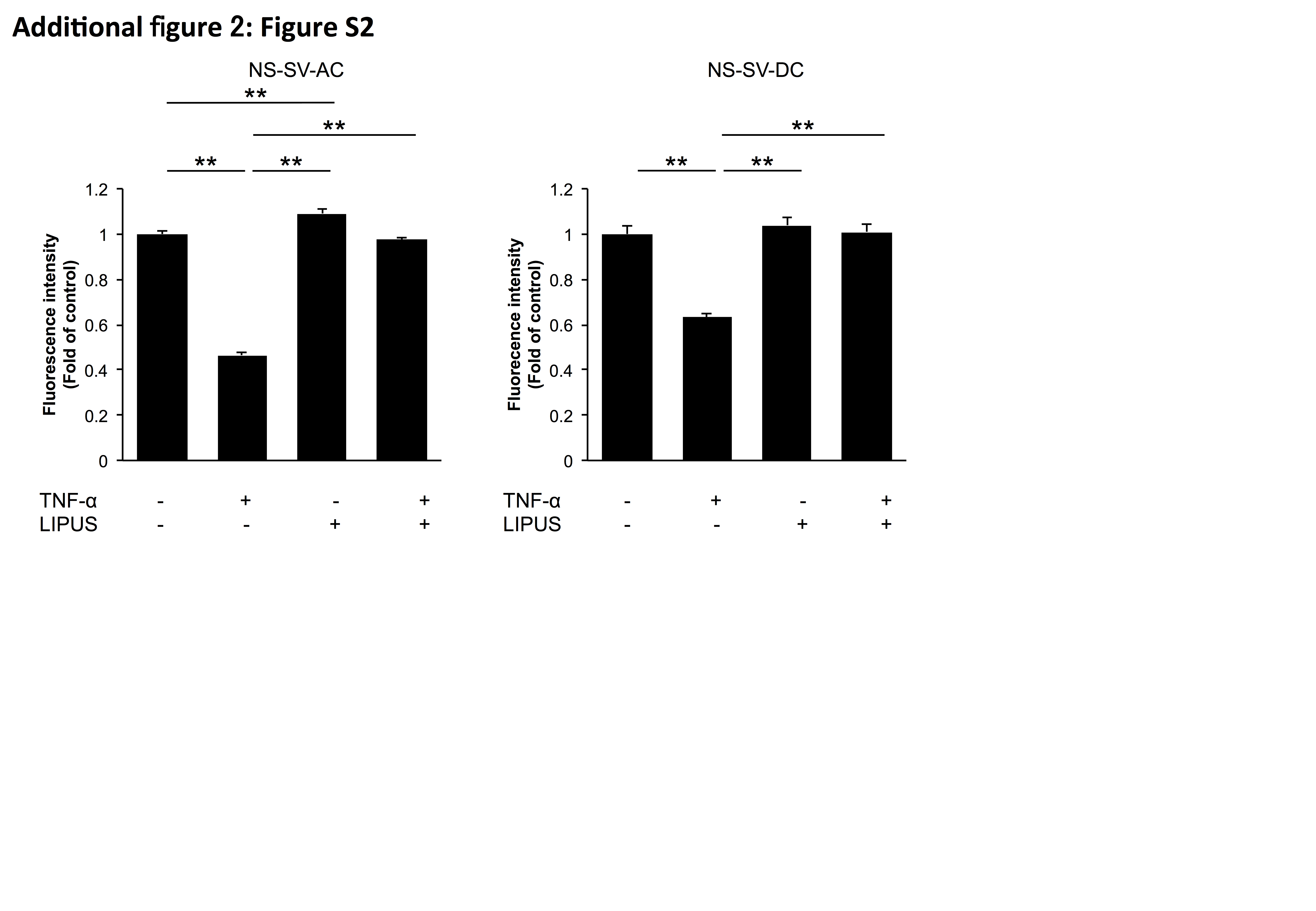

Supplement: Additional file 2: Figure S2. — Quantification of the fluorescence intensity in Fig. 1C. **p < 0.01 (n = 4). (TIFF 897 kb) [file 13075_2015_798_MOESM2_ESM.tiff]

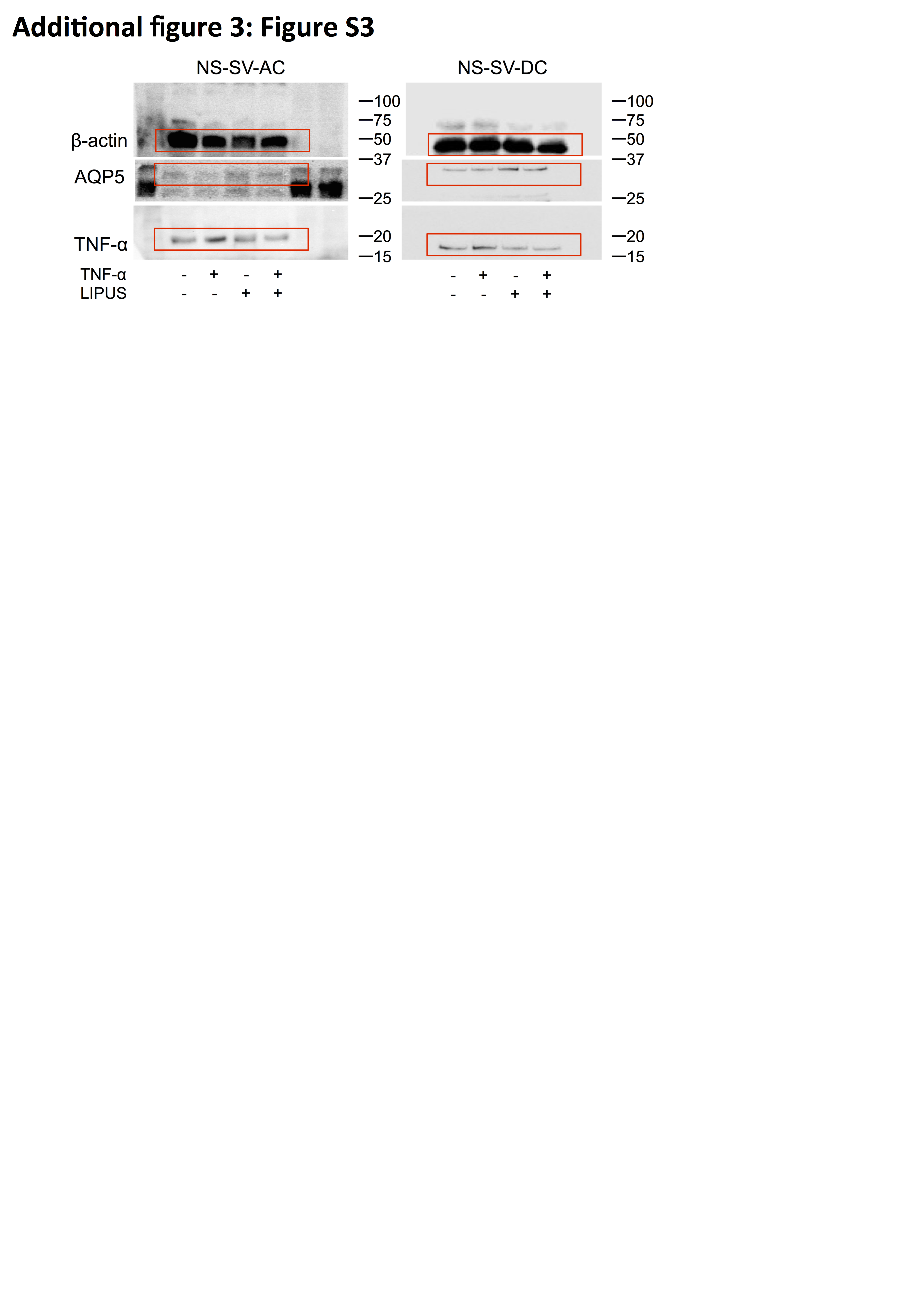

Supplement: Additional file 3: Figure S3. — Bands of Western blot analysis for the cropped images in Fig. 2c are provided in this file. (TIFF 1856 kb) [file 13075_2015_798_MOESM3_ESM.tiff]

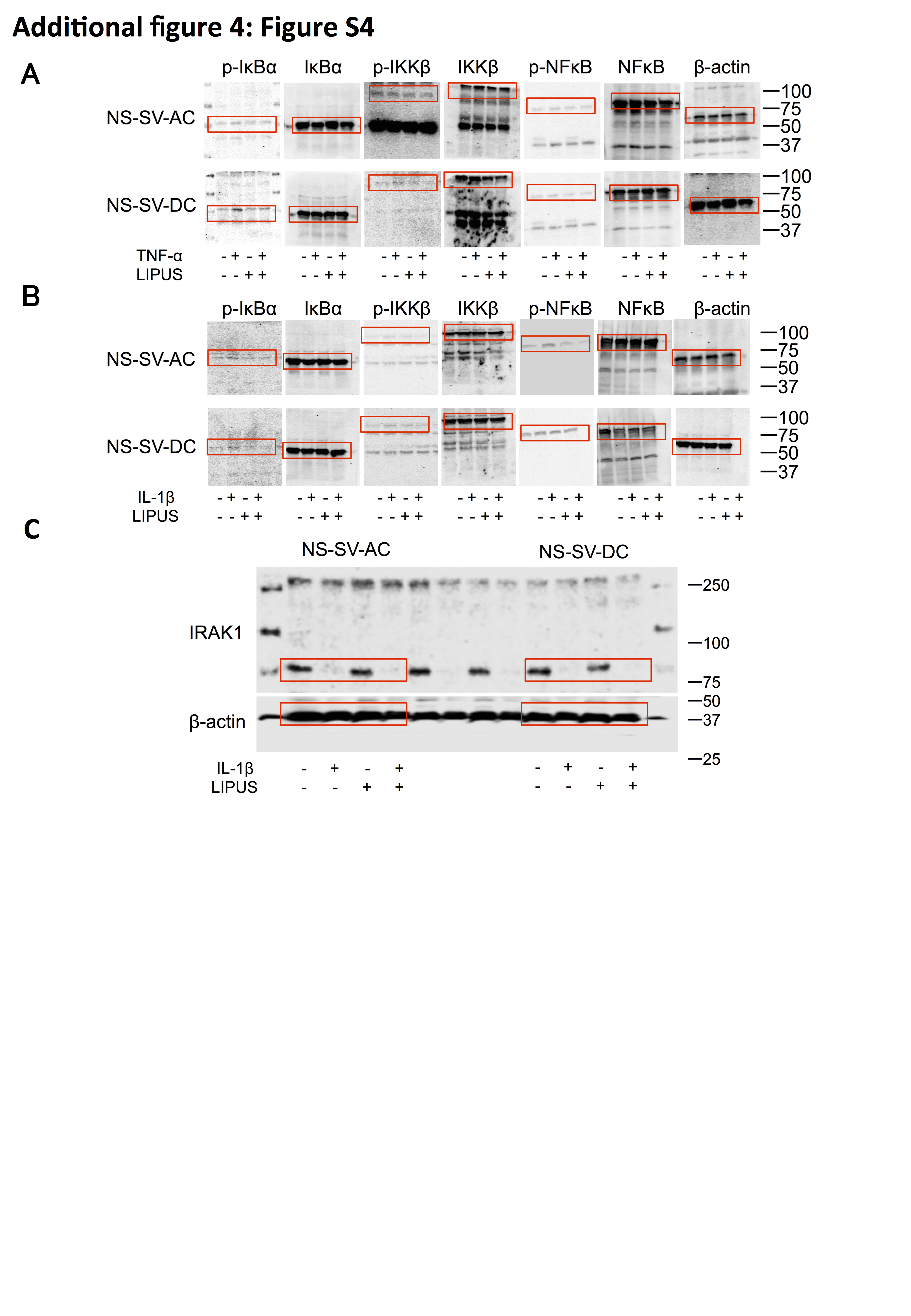

Supplement: Additional file 4: Figure S4. — Bands of Western blot analysis for the cropped images in Fig. 3a (A), 3b (B) and 3c (C) are provided in this file. (TIFF 5449 kb) [file 13075_2015_798_MOESM4_ESM.tiff]

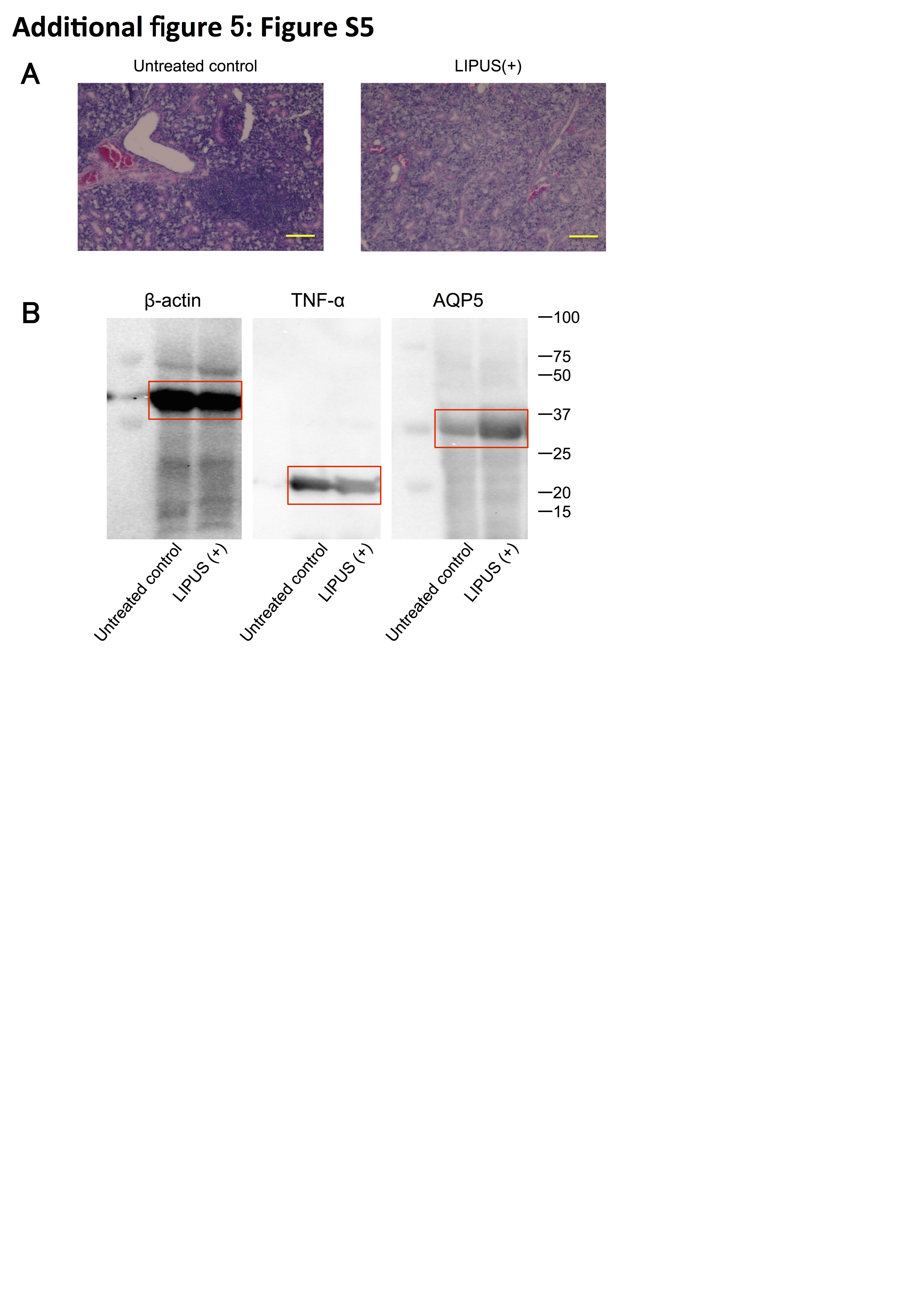

Supplement: Additional file 5: Figure S5. — (A) MRL/lpr mice treated with LIPUS exhibited a marked reduction in histological damage compared with untreated MRL/lpr mice. Scale bar = 100 μm. (B) Bands of Western blot analysis for the cropped images in Fig. 4e are provided in this file. (TIFF 5901 kb) [file 13075_2015_798_MOESM5_ESM.tiff]
